# Supplementary material for: Urinary 8-iso PGF2α and 2,3-dinor-8-iso PGF2α can be indexes of colitis-associated colorectal cancer in mice
Source: PLoS One. 2021 Jan 27;16(1):e0245292. doi: 10.1371/journal.pone.0245292 (PMC7840041; doi:10.1371/journal.pone.0245292)
Supplement: S1 Table — (DOCX) [file pone.0245292.s001.docx]

| Substance | Concentration (ng/ml in ethanol) |
| --- | --- |
| tetranor-PGEM-d_6_ | 200 |
| 6-keto-PGF_1α_-d_4_ | 2000 |
| TXB_2_-d_4_ | 200 |
| PGF_2α_-d_4_ | 200 |
| PGE_2_-d_4_ | 200 |
| PGD_2_-d_4_ | 200 |
| LTC_4_-d_5_ | 200 |
| LTB_4_-d_4_ | 200 |
| 5(S)HETE-d_8_ | 1000 |
| 12(S)HETE-d_8_ | 500 |
| 15(S)HETE-d_8_ | 200 |
| PAF C16-d_4_ | 200 |
| Oleoylethanolamide-d_4_ | 40 |

**S1 Table. The composition of internal standards mixture for comprehensive analysis.**
