# Supplementary material for: Urinary 8-iso PGF2α and 2,3-dinor-8-iso PGF2α can be indexes of colitis-associated colorectal cancer in mice
Source: PLoS One. 2021 Jan 27;16(1):e0245292. doi: 10.1371/journal.pone.0245292 (PMC7840041; doi:10.1371/journal.pone.0245292)
Supplement: S2 Table — (DOCX) [file pone.0245292.s002.docx]

| **S**ubstance | Concentration (ng/ml in ethanol) |
| --- | --- |
| tetranor-PGEM-d_6_ | 200 |
| tetranor-PGDM-d_6_ | 100 |
| 8-iso-PGF_2α_-d_4_ | 200 |
| 11-dehydro-TXB_2_-d_4_ | 100 |
| LTE_4_-d_5_ | 200 |

**S2 Table. The composition of internal standards mixture for absolute measurements.**
