# Supplementary material for: Urinary 8-iso PGF2α and 2,3-dinor-8-iso PGF2α can be indexes of colitis-associated colorectal cancer in mice
Source: PLoS One. 2021 Jan 27;16(1):e0245292. doi: 10.1371/journal.pone.0245292 (PMC7840041; doi:10.1371/journal.pone.0245292)
Supplement: S3 Table — (DOCX) [file pone.0245292.s003.docx]

| Comprehensive analysis | | |  |
| --- | --- | --- | --- |
| Step | Time (min) | Mobile phase A (%) | Mobile phase B (%) |
| 0 | 0 | 90 | 10 |
| 1 | 5 | 75 | 25 |
| 2 | 10 | 65 | 35 |
| 3 | 20 | 25 | 75 |
| 4 | 25 | 5 | 95 |
| 5 | 27 | 90 | 10 |
|  |  |  |  |
| Absolute measurement | | |  |
| Step | Time (min) | Mobile phase A (%) | Mobile phase B (%) |
| 0 | 0 | 90 | 10 |
| 1 | 1 | 90 | 10 |
| 2 | 9 | 70 | 30 |
| 3 | 17 | 5 | 95 |
| 4 | 21 | 90 | 10 |

**S3 Table. Gradient program for comprehensive analysis and absolute measurement.**
