# Supplementary material for: Urinary 8-iso PGF2α and 2,3-dinor-8-iso PGF2α can be indexes of colitis-associated colorectal cancer in mice
Source: PLoS One. 2021 Jan 27;16(1):e0245292. doi: 10.1371/journal.pone.0245292 (PMC7840041; doi:10.1371/journal.pone.0245292)
Supplement: S4 Table — Ion mode was selected fromnegative (-) or positive (+). (DOCX) [file pone.0245292.s004.docx]

| Substance | Ion mode | m/z | Elution time |
| --- | --- | --- | --- |
| tetranor-PGEM | - | 327→309 | 6.6 |
| tetranor-PGEM-d_6_ | - | 333→297 |  |
| tetranor-PGDM | - | 327→309 | 7.1 |
| tetranor-PGDM-d_6_ | - | 333→315 |  |
| 2,3-dinor-8-iso-PGF_2α_ | - | 325→237 | 11.9 |
| 8-iso-PGF_2α_ | - | 353→193 | 12.7 |
| 8-iso-PGF_2α_-d_4_ | - | 357→197 |  |
| 11-dehydro-TXB_2_ | - | 367→305 | 13.5 |
| 11-dehydro-TXB_2_-d_4_ | - | 371→309 |  |
| LTE_4_ | + | 440→189 | 14.5 |
| LTE_4_-d_5_ | + | 445→194 |  |

**S4 Table. Ion mode, m/z value and elution time of each substance for absolute measurement.**
