# Supplementary material for: Urinary 8-iso PGF2α and 2,3-dinor-8-iso PGF2α can be indexes of colitis-associated colorectal cancer in mice
Source: PLoS One. 2021 Jan 27;16(1):e0245292. doi: 10.1371/journal.pone.0245292 (PMC7840041; doi:10.1371/journal.pone.0245292)
Supplement: S5 Table — The values are shown as fold increase ± SE compared with the average of vehicle treated mice. DGLA, dihomo-γ-linoleic acid; AA, arachidonic acid; EPA, eicosapentaenoic acid; COX, cyclooxygenase; LOX, lipoxygenase; CYP, cytochrome P450; PG, prostaglandin; TX, thromboxane; LT, leukotriene; DiHETE, dihydroxyeicosatetraenoic acid; OxoEDE, oxoeicosadienoic acid; -, non-enzymatic oxidation. (DOCX) [file pone.0245292.s005.docx]

**S5 Table. Lipid metabolites detected in the urines of CAC mice model.**

| PUFA(Enzyme) | Precursor | Metabolite | Day 1 | Day 60 | p value |
| --- | --- | --- | --- | --- | --- |
| DGLA |  | 8-iso PGF_1β_ | 1±0.33 | 2.02±0.19 | 0.18 |
| AA (COX) | PGE_2_ | 20-OH PGE_2_ | 1±0.33 | 2.94±1.1 | 0.12 |
|  |  | 15-keto PGE_2_ | 1±0.26 | 0.80±0.17 | 0.52 |
|  |  | 13,14-dihydro-15-keto PGE_2_ | 1±0.37 | 1.92±0.66 | 0.30 |
|  |  | PGA_2_ | 1±0.27 | 0.43±0.08 | 0.052 |
|  |  | PGF_2α_ | 1±0.37 | 1.45 ±0.51 | 0.49 |
|  | PGF_2α_ | tetranor-PGFM | 1±0.26 | 2.37±0.67 | 0.07 |
|  |  | 6-keto PGF_1α_ | 1±0.28 | 2.02±0.57 | 0.13 |
|  | TXA_2_ | TXB_2_ | 1±0.42 | 2.06±0.52 | 0.14 |
|  |  | 11-dehydro TXB_2_ | 1±0.35 | 1.78±0.42 | 0.19 |
|  | PGD_2_ | 11-beta PGF_2α_ | 1±0.41 | 1.10±0.35 | 0.84 |
| AA (LOX) | LTB_4_ | 20-OH LTB | 1±0.17 | 1.17±0.49 | 0.75 |
|  |  | 20-carboxy LTB_4_ | 1±0.15 | 1.72±0.42 | 0.13 |
|  |  | 18-carboxy-dinor LTB_4_ | 1±0.27 | 4.44±1.1 | **0.009** |
|  | LTC_4_ | LTD_4_ | 1±0.25 | 0.48±0.16 | 0.10 |
|  |  | 11-trans LTE_4_ | 1±0.28 | 1.30±0.29 | 0.62 |
| AA (-) |  | 8-iso-15(R) PGF_2α_ | 1±0.32 | 1.47±0.42 | 0.41 |
| EPA (COX) |  | PGE_3_ | 1±0.42 | 0.27±0.07 | 0.19 |
| EPA (CYP) |  | 17,18-DiHETE | 1±0.14 | 2.80±0.86 | **0.04** |
| EPA (-) |  | 8-iso PGF_3α_ | 1±0.33 | 1.95±0.51 | 0.14 |
